# An antimicrobial thiopeptide producing novel actinomycetes *Streptomyces terrae* sp. nov., isolated from subsurface soil of arable land

**DOI:** 10.1093/femsmc/xtad014

**Published:** 2023-08-10

**Authors:** Stanzin Choksket, Mahaldeep Kaur, Anil Kumar Pinnaka, Suresh Korpole

**Affiliations:** CSIR-Institute of Microbial Technology, Sector 39A, Chandigarh-160036, India; CSIR-Institute of Microbial Technology, Sector 39A, Chandigarh-160036, India; CSIR-Institute of Microbial Technology, Sector 39A, Chandigarh-160036, India; CSIR-Institute of Microbial Technology, Sector 39A, Chandigarh-160036, India

**Keywords:** *Streptomyces*, genome, ANI, thiopeptide, berninamycin, BGC

## Abstract

An antimicrobial producing Gram-positive, aerobic, nonmotile, and filamentous actinobacterial strain SKN60^T^ was isolated from soil The isolate exhibited 99.3% and 99.0% identity with *Streptomyces laurentii* ATCC 31255^T^ and *S. roseicoloratus* TRM 44457^T^, respectively, in 16S rRNA gene sequence analysis. However, the genome sequence displayed maximum ANI (88.45%) and AAI (85.61%) with *S. roseicoloratus* TRM 44457^T^. Similarly, the dDDH showed 33.7% identity with *S. roseicoloratus* TRM 44457^T^. It formed a cluster with *S. roseicoloratus* TRM 44457^T^ and *S. laurentii* ATCC 31255^T^ in phylogenomic tree. Cell wall analysis revealed the presence of diphosphatidylglycerol, phosphatidylethanolamine, and phosphatidylcholine as major polar lipids and diaminopimelic acid as diagnostic diamino acid. Major fatty acids were iso-C_15:0_, anteiso-C_15:0_, and iso-C_16:0_. The G+C content was found to be 72.3 mol%. Genome sequence analysis using antiSMASH database showed occurrence of a thiopeptide biosynthesis gene cluster with 94% similarity to berninamycin from *S. bernensis* UC5144. The mass of 1146 Da is identical with berninamycin. But subtle differences observed in leader peptide sequence of thiopeptide and berninamycin. Notably, *S. bernensis* is not validly reported and thus SKN60^T^ is the only strain containing berninamycin BGC as no other phylogenetic relative had it. Additionally, strain SKN60^T^ differed in phenotypic and genetic characteristics with all phylogenetic relatives of the genus *Streptomyces*. Therefore, it is proposed as a novel species with the name *Streptomyces terrae* sp. nov. strain SKN60^T^ (=MTCC 13163^T^; = JCM 35768^T^).

## Teaser

Thiopeptide producing *Streptomyces terrae* SKN60T sp. nov.

## Introduction

Actinomycetes constitute a major portion of soil microbial biomass. *Streptomyces* is one of the important genera of *Actinomycetota* phylum and contains the largest number of species, which differ mostly in their physiology, biochemical, and morphological features. Members of the genus *Streptomyces* are well known for the antibiotic production (Neha et al. 2013). The genus *Streptomyces* contains Gram-positive filamentous bacterial species with high G+C content (Skerman et al. 1989). Currently, it contains more than 800 species isolated from aquatic and terrestrial ecosystems, indicating their ubiquitous nature. *Streptomyces* species exhibit a mycelial life cycle with the formation of aerial mycelium containing single spore or spore chains (Zhang et al. 2013). The spores are grown as nonseparate mycelium with branching hyphal filaments under optimal environmental conditions. It is best studied genus to display such modifications and a complex life cycle (Flardh and Buttner 2009). Members of the genus exhibit an extensive secondary metabolism and produce an array of bioactive substances, including antibiotics of medical importance (Challis and Hopwood 2003). The production of secondary metabolic bioactive substances is usually growth phase dependent and observed during the shifting of mycelium to the sporulation phase (van Wezel and McDowall 2011, Hwang et al. 2014). Approximately, two-thirds of all available naturally derived bioactive substances including anticancer, anthelmintic, antibacterial, and antifungal compounds were reported to produce by the various species of the genus *Streptomyces* (Uyeda et al. 2001, Procopio et al. 2012, Ahmad et al. 2017). In fact, some species have been employed as biocontrol agents in agriculture (Wang et al. 2018). Consequently, these bacteria are of major importance to the biotechnology and pharmaceutical industry. Furthermore, identification of antimicrobial biosynthesis potential strain discovery has been increased with the advances in genomics, particularly with the deposition of whole genome and draft genome sequences in public databases. Genome mining has helped to identify the exact constituents of secondary metabolite producers and revealed the biosynthetic potential of different bacteria (Udwary et al. 2011, Bauman et al. 2021). It also provides an opportunity for genome mining combined with metabolic profiling, which results in the heterologous expression of antimicrobial compounds (Yamada et al. 2015, Chen et al. 2017). Berninamycin is an example for genome mining-based heterologous expression (Malcolmson et al. 2013). It is identified as pyridine containing thiopeptide that shows post-translational modifications upon synthesis. The modifications include formation of dehydroalanine/dehydrobutyrines and azole/azoline rings (Bycroft and Gowland 1978). These antimicrobial peptides are an emerging class of antibiotics against drug-resistant Gram-positive bacteria. Since species of the genera *Streptomyces* and *Nocardia* are primarily reported to produce antimicrobials (Jiang et al. 2023, Bauman et al. 2021, Bagley et al. 2005) in this study, we have made an attempt to isolate and identify such antimicrobial substances including antimicrobial peptide producing strains of these genera.

## Materials and methods

### Soil sample collection and isolation of actinomycetes

Soil sample was collected from subsurface (depth of 20 cm) environment of an arable land in Chandigarh and processed immediately to screen microorganisms exhibiting antimicrobial activity. Briefly, 1 g of soil was serially diluted in 10 ml of sterile saline and 100 µl aliquots of each dilution (10^−2^–10^−4^) were inoculated on to starch casein agar (Himedia, India) plates. The plates were incubated at 30ºC for 3–4 days and observed after every 24 h time interval for antimicrobial producing strains. Colonies showing inhibitory zone in their periphery were picked up and streaked on *Streptomyces* agar (Himedia) for further purification, and stored as glycerol stocks in −70ºC deep freezer until further use. A strain designated as SKN60 was selected for detailed studies.

### Morphological and physiological characterization

The morphological features were checked by growing the strain aerobically at 30ºC for 2–3 days on various International *Streptomyces* Project (ISP) media (Shirling and Gottlieb 1966) including tryptone yeast extract agar (ISP1), yeast malt agar (ISP2), oat meal agar (ISP3), inorganic salt starch agar (ISP4), glycerol asparagine agar (ISP5), peptone yeast extract iron agar (ISP6), tyrosine agar (ISP7), and *Streptomyces* agar. The aerial and submerged mycelium was observed for color and pigment production. Strain morphology was observed through a phase contrast microscope and sporulation pattern was observed by allowing strain growth onto coverslips inserted into agar medium. Physiological studies were determined at different conditions like temperature (20–40ºC), pH (2–10), and salt concentrations (0%–8%) (Cowan and Steel 1965). Different biochemical tests including the ability to utilize different carbon substrates were studied as per the recommended methods (Smibert and Krieg 1994).

### Chemotaxonomic characterization

Lyophilized cell biomass of strain SKN60^T^ was used for different chemotaxonomic analysis. The fatty acid methyl esters were analyzed by Sherlock Microbial Identification System using version 6.1 (Sasser 1990). Total cellular polar lipids, menaquinone, and peptidoglycan analysis were performed by thin layer chromatography (TLC) analyses (Minnikin et al. 1984, Schumann 2011). The extracted polar lipids were analyzed using two-dimensional TLC. Different staining reagents, including molybdatophosphoric acid, ninhydrin, molybdenum blue, and α-naphthol reagents, were employed to detect total lipids, aminolipids, phospholipids, and glycolipids, respectively.

### 16S rRNA gene sequence analysis

The genomic DNA of SKN60^T^ was extracted using Zymo DNA isolation kit and used for 16S rRNA gene amplification. Briefly, 16S rRNA gene was amplified using the primers 27F and 1492R and 5x firepol master mix (SolisBioDyne, Estonia) in a 50 µl reaction volume. The amplified product was run using 1% agarose gel and purified using HiYield™ Gel/PCR DNA mini kit (Real Biotech Corporation). The purified amplicon was sequenced using three internal primers including 27F, 786F, and 1492R (10 µl reaction volume) and the sequencing was performed on an ABI Prism 3700 automatic DNA sequencer (Applied Biosystems). The nucleotide sequence file (.ab1) files were analyzed using FinchTV version (1.4.0) software and assembled manually. The consensus sequence was used for BLAST sequence similarity search on EzTaxon server (Chun et al. 2007). All closely related sequences were retrieved in fasta format and aligned with SKN60^T^ sequence using CLUSTAL_W (Thompson et al. 1994). The phylogenetic tree was constructed by neighbor-joining algorithm using MEGA software version 7.0 (Tamura et al. 2011). Kimura two-parameter was used to calculate evolutionary distances and the tree topologies were evaluated by bootstrap analyses with 1000 replications.

### Genome sequencing and analyses

The genome sequence of SKN60^T^ was produced using illumina-HiSeqX10 at Agrigenome, India. The total genome coverage was 150X and library was prepared using Paired-end and Mate pair. The library for genome sequencing was prepared using Nextera DNA XT library preparation Kit (15031942). Fastq quality check of the reads was performed using FastUniq (Xu et al. 2012). De-novo assembly was performed using ABySS (1.2.10), SPAdes (3.1.30), and Velvet (1.2.150) (http://www.usadellab.org/cms) version 3.13 (Bankevich et al. 2012). Quality assessment of the genome assemblies was performed using QUAST 4.0 version (Gurevich et al. 2013), which provides better statistics for velvet assembly and the presence of assembled genes were checked using BUSCO V2 (Simao et al. 2015). Genome annotation of strain SKN60^T^ was performed using Rapid annotation using subsystem technology (RAST) server (https://rast.nmpdr.org/). Prediction of protein coding genes was performed using BLASTX search against UniProt database and Prokka, v.1.14.6 (Seemann 2014). The 16S rRNA gene was extracted using RNAmmer tool (Lagesen et al. 2007). The whole genome sequence (WGS) shotgun data of the strain SKN60^T^ was deposited in GenBank. Further, the WGS of *Streptomyces* sp. SKN60^T^ was uploaded to the Type (Strain) Genome Server (TYGS) for conducting a taxonomic based analysis of the whole genome (Meier-Kolthof and Göker 2019). Genome blast distance phylogeny (GBDP) was used to compare the SKN60^T^ genome with the type strains. Intergenomic distances were accurately estimated using the ‘trimming’ algorithm and the d5 distance formula. These intergenomic distances were used to construct a balanced minimum evolution tree using FASTME 2.1.4 with branch support determined through 100 pseudo-bootstrap replicates (Henz et al. 2005). Biosynthetic gene cluster (BGC) encoding for the production of secondary metabolites was analyzed using antiSMASH 7.0 database tool (Blin et al. 2019). Whole genome-based comparison of SKN60^T^ was analyzed using bioinformatic tools like CGView Comparison Tool (CCT) (http://stothard.afns.ualberta.ca/cgview_server) and Proksee (https://proksee.ca/) for circular map representations.

### Pan and core-genome analysis

Pan-genome analysis of SKN60^T^ was performed using a method of Vernikos et al. (2015). The pan-genome analysis was performed with genome sequence of the type strains of various species of the genus *Streptomyces* downloaded from the NCBI database. The pan-genome studies were performed using bacterial pan-genome analysis (BPGA) pipeline (Chaudhari et al. 2016). Average nucleotide identity (ANI) and average amino acid identity (AAI) with the close relatives were determined using ANI/AAI matrix service using Kostas lab (http://enve-omics.ce.ga?>020tech.edu/g-matrix/). Genetic relatedness based on digital DNA–DNA hybridization (dDDH) was calculated using genome-to-genome distance-based calculator (GGDC, www.dsmz.de/services/online-tools).

### Antimicrobial activity determination

Growth curve analysis of strain SKN60^T^ was performed using *Streptomyces* broth to detect antimicrobial production. Cell-free fermented broth (CFB) collected at different time intervals was checked for activity against different test strains procured from Microbial Type Culture Collection and Gene bank (MTCC), Chandigarh. Briefly, 50 ml of culture medium was inoculated with a loopful of inoculum and incubated at 30°C, 180 rpm and CFB fractions collected after every 24 h were tested for antimicrobial activity against test strains and cell biomass also determined. Gram-positive bacteria including *Bacillus subtilis* (MTCC 121), *Staphylococcus aureus* (MTCC 1430), *Micrococcus luteus* (MTCC 106), and Gram-negative bacteria including *Pseudomonas aeruginosa* (MTCC 1934), *Vibrio cholera* (MTCC 3904), and *Escherichia coli* (MTCC 1610) were used as test strains in this study. Overnight grown culture (50 µl) was spread on a nutrient agar plate. Subsequently, wells were made using a cork-borer and 100 µl of CFB collected at different time intervals was added to the wells. Plates were incubated overnight under optimal growth conditions to measure the zone of inhibition.

### Extraction and purification of antimicrobial compound

The production of antimicrobial compound was carried out by inoculating 1% of an overnight grown culture of strain SKN60^T^ (grown in *Streptomyces* broth) in 1000 ml of nutrient broth (NB) medium and incubated at 30°C for a period of 96 h at 180 rpm. The resulting culture medium was centrifuged at 7000 rpm, 4°C for 30 min. The harvested CFB was sterilized using polyether sulfone (PES) membranes with a pore size of 0.2 µm. The antimicrobial compound was extracted by solvent extraction method using ethyl acetate: CFB in equal volumes. The organic layer was collected and evaporated in a rotatory evaporator (Buchi). The active fraction was finally suspended in 100% methanol. The compound was analyzed on a silica plate using TLC (in duplicates). The organic solvent mobile phase employed was chloroform: methanol: water (65:45:4). The processed TLC plate was dried inside a fume hood and cut into two sections. One strip was sprayed with ninhydrin reagent to detect the peptide. Another strip was used to check the antimicrobial activity against *S. aureus* MTCC 1430 (∼10^6^ cells ml^−1^) in a bioautography assay. Briefly, the strip was placed on a cultured nutrient agar plate and allowed to diffuse at 4°C for 1 h. The TLC strip was removed after diffusion and plates were kept at 37°C to observe its inhibitory activity. The antimicrobial compound was purified using reverse phase high-performance liquid chromatography (HPLC) on 1260 infinity instrument (Agilent Technologies, USA) using a semipreparative C18 column. The mobile phase consisted of solvent system A [HPLC grade water with 0.12% trifluoroacetic acid (TFA)] and solvent system B (HPLC-grade acetonitrile (ACN) with 0.1% TFA) and was monitored via UV detector at 220 and 280 nm. The purified fraction was collected manually and used for further studies.

### Mass spectrometry

The ESI-Q-TOF mass spectrometry was carried out using Agilent 6550 (LC 1290 infinity) system coupled with 6550 Q-TOF (Agilent Technologies). Mobile phase consisted of 0.1% formic acid in water (solvent A) and acetonitrile with 0.1% formic acid (solvent B). The eluted sample was exposed to positive ionization mode at fragmentor voltage 100 V. The spectrum was acquired in low m/z range. The intact mass of HPLC purified peptide was determined by matrix-assisted laser desorption ionization (MALDI). The peptide (0.7 mg ml^−1^) was mixed with α-cyano-4-hydroxycinnamic acid matrix in a ratio of 1:1 and loaded on a stainless steel MALDI target plate. The sample was then processed after air drying for MALDI analysis (Bruker, Microflex).

### MIC and killing kinetic determination assay

The method of Gupta et al. (2019) was followed to determine minimum inhibitory concentration (MIC) of the purified peptide using 96-well plate assay. MIC is defined as 90% growth inhibition of the test strains. Additionally, killing kinetics of the antimicrobial substance from SKN60^T^ was determined against various Gram-positive test strains. Briefly, cells grown to the logarithmic phase were collected by centrifugation and subsequently washed with phosphate buffer saline (PBS, Gibco). The final cell count was adjusted to an OD_600_ 0.3–0.4. The cells were exposed to different concentrations of the active compound for various time intervals and aliquots of the suspensions were spreaded on plates. Time–kill kinetics were expressed as CFU counts. Untreated cells were used as a control to check CFU count.

### TEM sample preparation

TEM was performed to elucidate the mechanism of antimicrobial activity resulting upon treatment of *S. aureus* (MTCC 1430) with different concentrations of antimicrobial substance. Untreated control also processed in parallel (Gupta et al. 2019). Briefly, cells were suspended in 1X PBS to obtain the cell density of 10^8^ CFU ml^−1^ and treated with different concentrations (2X and 5X MIC) of a peptide. Samples were subsequently subjected to negative stain with 0.1% (w/v) Sodium phosphotungstate (PTA, Sigma) for 1 min using carbon-coated copper grid (300 mesh, Polybioscience) and cells were observed using a transmission electron microscope (TEM) at a resolution of 0.1–0.5 µm range (Raje et al. 2006).

### Haemolysis

Haemolysis assay was performed using blood of New Zealand white rabbit, which was collected from iCARE-CSIR-IMTech. Freshly collected blood was centrifuged and cells were reconstituted using 1X PBS. The number of RBCs was counted using a hemocytometer and the final cell number was adjusted to 2 × 10^8^ cells ml^−1^. About 5% of RBCs were treated with different SKN60^T^ peptide concentrations and incubated in a CO_2_ (5%) incubator for various time intervals at 37°C. PBS and 1% tritonX 100 were considered as negative and positive controls, respectively. Lysis of RBCs was measured using a spectrophotometer with absorbance at 541 nm for different time intervals to observe lysis of RBCs (Singh et al. 2014).

## Results

### Isolation and characterization

**Figure 1.**
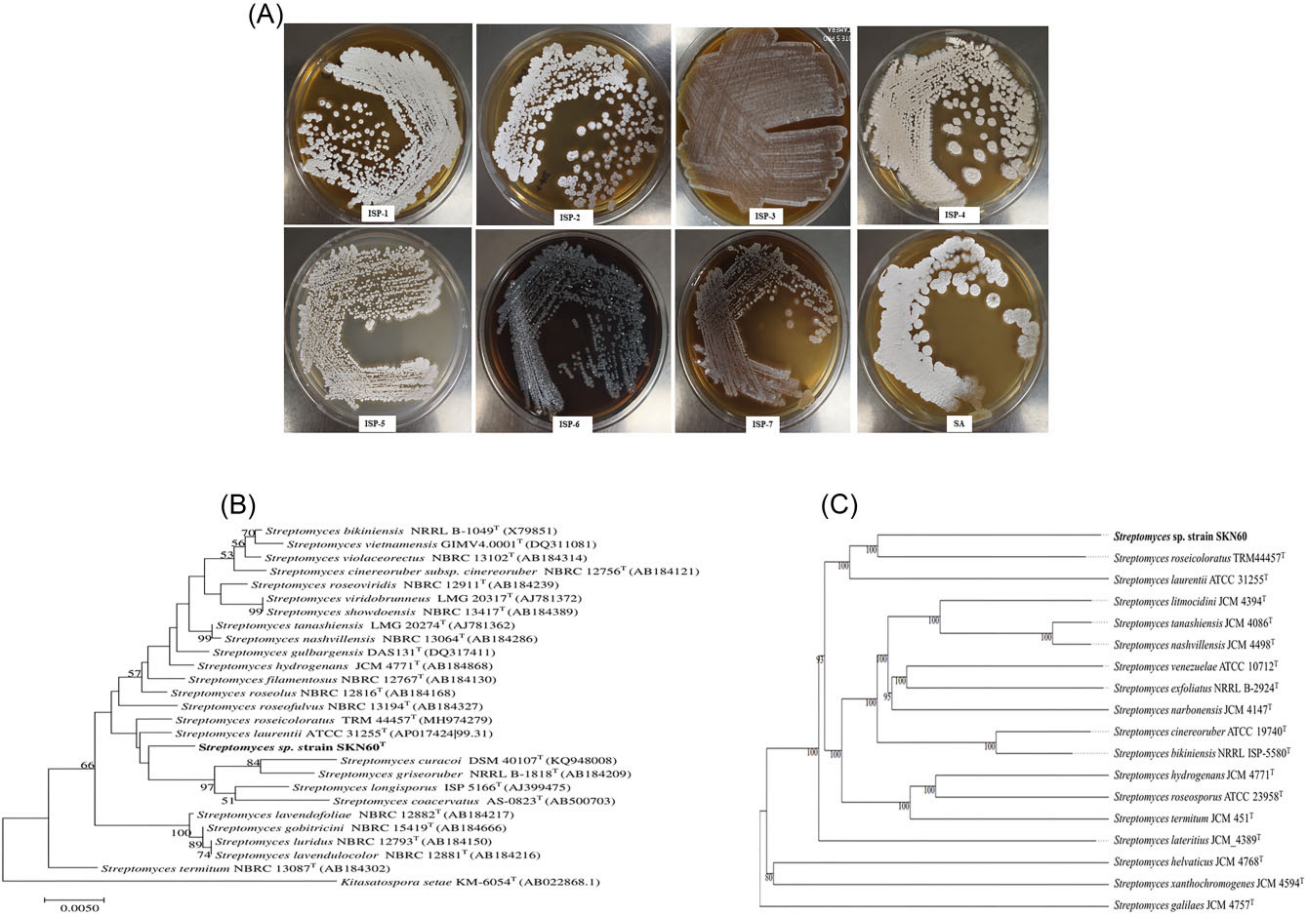
Phenotypic and genetic characterization of strain SKN60^T^. **(A)** Cultural characteristics of SKN60^T^ strain on ISP-1 to ISP-7 media and *Streptomyces* agar showing substrate mycelium, aerial mycelium, sporulation, and secretion of soluble pigment. **(B)** Phylogenetic relation of strain SKN60^T^ with close relatives based on neighbor-joining method using 16S rRNA gene sequences. Bootstrap values > 50% are displayed on their relative branches. Kimura 2_parameter method was considered to measure the evolutionary distances and gamma distribution was set to 0.5 **(C)** Intergenomic distances based phylogenomic tree showing relation of strain SKN60^T^ with other closest relatives. It was performed using GBDP. The analysis showed formation of a distinct clade of strain SKN60^T^ with *Streptomyces roseicoloratus* TRM 44457^T^.

While screening rhizosphere soil samples for microorganisms possessing antimicrobial activity, a strain designated as SKN60 with an inhibitory zone was isolated and studied in detail. Strain SKN60^T^ showed good growth on all media including ISP1–ISP7 and *Streptomyces* agar. It produced aerial and submerged mycelium. Aerial mycelium was off-white on ISP1, ISP2 and *Streptomyces* agar media, and it was cream in color on all other media used for cultivation (Fig. 1A). Soluble pigment production was observed when it was grown on ISP6 medium but no diffusible pigment production was observed in any other media. Substrate mycelia were cream-colored in all media except ISP4, ISP6, and ISP7, which showed brownish color. Cream sporulation was observed on ISP1, ISP2, ISP4, ISP5, and *Streptomyces* agar. *Streptomyces* agar was used to grow strain for further characterization. Strain SKN60^T^ is a mycelium forming Gram-positive actinobacteria. It showed positive reactions to nitrate reduction and oxidase enzyme tests. It showed a negative reaction for indole, urease, citrate, arginine dehydrolase, methyl red, and Voges–Proskauer tests. Hydrolysis of esculin, casein and starch were observed. Hydrolysis of Tween 20 and 40 was negative but weakly positive for Tween 60 and 80. It showed a negative reaction to catalase and gelatine tests. It was able to utilize arabinose, sucrose, dulicitol, galactose, lactose, mannose, mannitol, melibiose, d-myo inositol, raffinose, rhamnose, sorbitol, salicin, trehalose, and xylose as carbon source. Growth was observed up to 40ºC temperature with optimum growth at 30ºC and pH tolerance up to 10. It was observed that strain SKN60^T^ was able to tolerate 8% NaCl concentration. Further, strain SKN60^T^ differed in biochemcial and physiological properties with closet relatives (Table S1, Supporting Information). The fatty acid profile showed presence of (%) iso-C_10:0_ (10.9), iso-C_14:0_ (7.2), iso-C_15:0_ (10.9), anteiso-C_15:0_ (11.2), iso-C_16:0_ (8.4), anteiso-C_17:0_ (3.0), iso-C_17:1_ω5c (6.1), and iso-C_17:0_ 3-OH (7.5) as major fatty acids. The polar lipid analysis using two-dimensional TLC and subsequent differential staining showed presence of diphosphatidylglycerol (DPG) and phosphatidylethanolamine (PE) as major polar lipids. The presence of phosphatidylcholine (PC), phosphatidylinositol (PI), and an unidentified phospholipid was also observed in minor quantities (Figure S1, Supporting Information). The menaquinone analysis showed presence of MK-9. The peptidoglycan analysis of cell wall lysate using TLC showed diaminopimelic acid (DAP) as a characteristic cell wall diamino acid.

### 16S rRNA gene phylogeny

Taxonomic affiliation of strain SKN60^T^ was established using EzBioCloud database blast search of 16S rRNA gene sequence (1404 bp, NCBI accession number MZ642351) and closely related phylogenetic neighbors were identified. Further, 16S rRNA gene sequence retrieved from genome sequence (1532 bp) was relatively longer and identical with sequence obtained from Sanger method, and thus, it was used for similarity search and phylogeny. The maximum sequence identity was observed with type strains of *Streptomyces laurentii* ATCC 31255^T^ (99.31%), *S. roseicoloratus* TRM 44457^T^ (99.03%), *S. showdoensis* NBRC 13417^T^ (98.47%), and *S. vietnamensis* GIMV4.0001^T^ (98.31%). The phylogenetic tree constructed using neighbor-joining phylogenetic analysis showed strain SKN60^T^ forming a cluster with closest phylogenetic relatives in a distinct clade (Fig. 1B). Indeed, phylogenetic analysis revealed that strain SKN60^T^ formed a monophyletic cluster with type strains of *S. roseicoloratus* TRM 44457^T^ and *S. laurentii* ATCC 31255^T^.

### Genome sequence analyses

The WGS of *Streptomyces* sp. strain SKN60^T^ contained 7.8 Mb assembled in 87 contigs (with pegs) with N50 contig size of 312 247 bp. The whole genome was submitted to NCBI under accession number JAHTMW000000000. Sequence based GC content was 72.3 mol%. The draft genome of strain SKN60^T^ was annotated using the prokaryotic genome annotation pipeline (PGAP, NCBI) and RAST analysis revealed 7396 CDS, 73 tRNA, 3 rRNA, 10 L50, and 312 247 N50. The genome of strain SKN60^T^ was employed for ANI calculation against 30 closely related genomes using Kostas lab server. None of the genomes of type strains compared with SKN60^T^ showed more than 95% ANI value. However, ANI analysis of genome sequences displayed maximum identity with *S. roseicoloratus* TRM 44457^T^ (88.45%), *S. showdoensis* NBRC 13417^T^ (87.02%), *S. laurentii* ATCC 31255^T^ (86.42%), and *S. vietnamensis* GIMV4.0001^T^ (85.91%) (Figure S2A, Supporting Information). Similarly, dDDH values also shown in descending order 33.7%, 30.7%, 29.2%, and 28.3% with its closest strains *S. roseicoloratus* TRM 44457^T^, *S. showdoensis* NBRC 13417^T^, *S. laurentii* ATCC 31255^T^, and *S. vietnamensis* GIMV4.0001^T^, respectively (Figure S2B, Supporting Information). The descending order of AAI values with closely related species showed 85.61%, 83.52%, and 80.71% identity with *S. roseicoloratus* TRM 44457^T^, *S. showdoensis* ATCC 15227^T^, and *S. laurentii* ATCC 31255^T^, respectively (Figure S2C, Supporting Information). The phylogenomic tree derived from intergenomic distances calculated using GBDP on TYGS server showed a distinct clade containing strains SKN60^T^ and *S. roseicoloratus* TRM 44457^T^ in a monophyletic cluster along with type strain of *S. laurentii* ATCC 31255^T^ (Fig. 1C).

**Figure 2.**
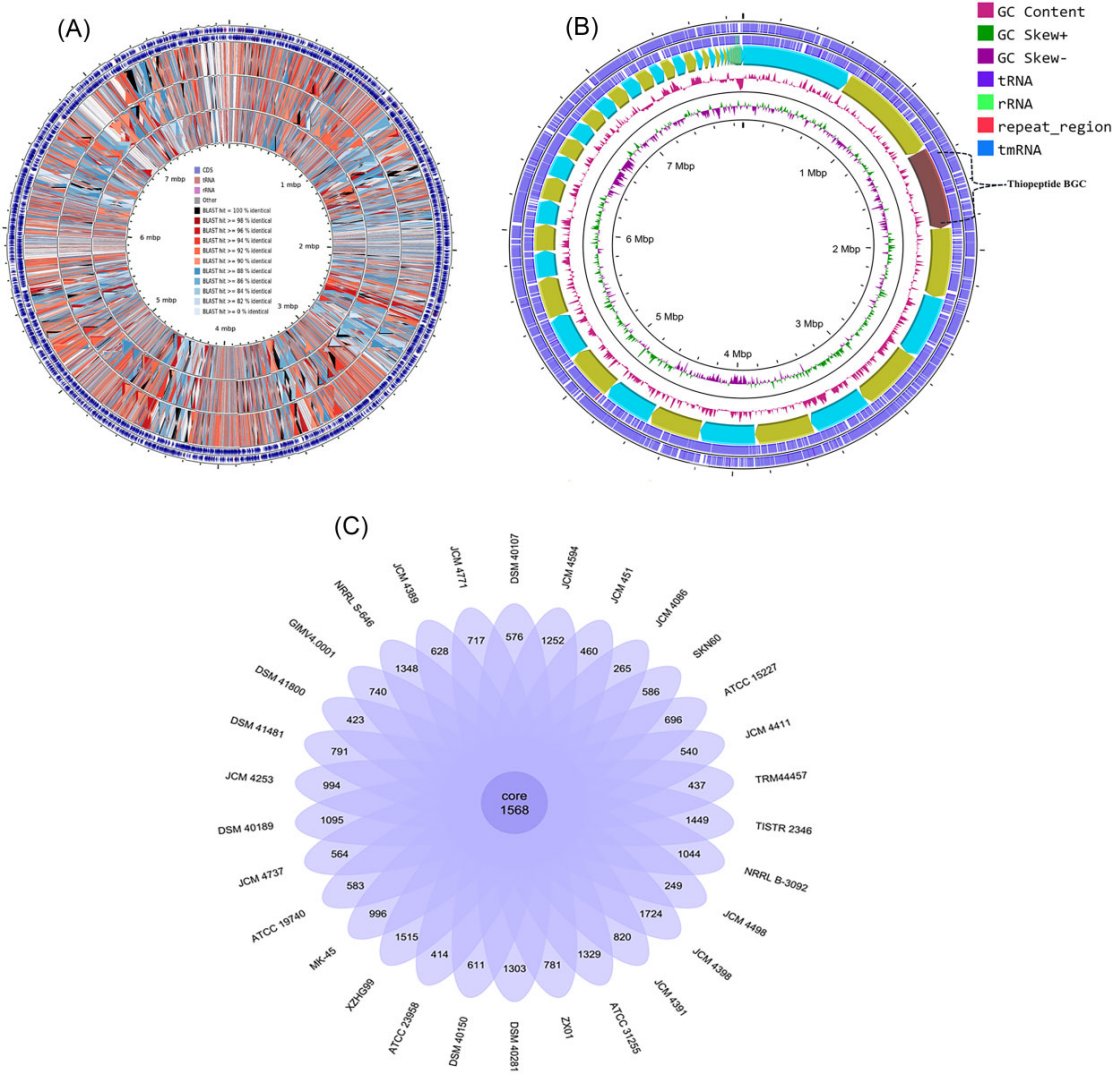
Genome comparative analyses of strain SKN60^T^ with close phylogenetic relatives. **(A)** Circular plot of genome comparison among the phylogenetic relative strains using CGView comparison tool (CCT). Outer most rings showing sense and antisense CDS followed by three rings showing blast hit comparison results outer to inner, SKN60^T^ vs. TRM 44457^T^, SKN60^T^ vs. ATCC 15227^T^, and SKN60^T^ vs ATCC 31255^T^) **(B)** Proksee based of strain SKN60^T^ showing different rings indicating the presence CDS (sense and antisense) in the outer most region, followed by different contigs. The contig which is brown in color is the berninamycin encoding region of SKN60^T^ peptides. The GC content, GC skew (positive and negative), tRNA, rRNA, repeated region, and tmRNA regions are also shown in the central portion of the circular map. **(C)** Flower pot diagram representation of the strain SKN60^T^ along with its closest strains showing BPGA for core, accessory, and unique gene distributions.

The high number of coding sequences (CDS) is accounted for the large genome size and functionally categorized CDSs have been assigned to cluster of orthologous groups using BASys platform. Graphical maps were generated using CCT to display the percentage blast similarities between the genomes of strain SKN60^T^ and those of *S. roseicoloratus* TRM 4457^T^, *S. showdoensis* ATCC 15227^T^, and *S. laurentii* ATCC31255^T^ for comparison purposes. The sense and antisense strands of genome are represented by the outermost two rings colored in blue hues, while the percentage of sequence similarity based on blast hits is displayed through coloring in the graphical representation (Fig. 2A). The three inner rings displayed areas of sequence similarity between CDS translation of the strain SKN60^T^ genome and those of *S. roseicoloratus* TRM 4457^T^, *S. showdoensis* ATCC 15227^T^, and *S. laurentii* ATCC 31255^T^, respectively, identified through BLAST comparisons. Different colors represent the percentage of blast hit identification, ranging from 100% to 0% in the graphical display. The genome sequence of strain SKN60^T^ was visualized in right order using Proksee server. The analysis displayed distinct rings in the outermost region indicating the presence of CDS in both the sense and antisense orientations, followed by various contigs. One of the contigs, depicted in brown, was identified as the berninamycin encoding BGC region in strain SKN60^T^ (Fig. 2B). The pan-genome analysis in terms of protein coding genes of 30 *Streptomyces* species genomes revealed 46 047 ortholog clusters from 162 482 total genes that comprised pan-genome. The sum of unique genes was clustered into 24 930 gene clusters. The core-genome contained 1568 gene clusters (Fig. 2C). The increase in total genomes decreased core-genome size, which is stabilized with about 1580 genes after the addition of 27th genome indicating the closure of core-genome. The core-pan plot revealed increase in pan-genome with the addition of new genomes, and therefore, it is considered as an open pan-genome (Figure S3, Supporting Information). The core and accessory gene pools were mainly associated with carbohydrate, nucleotide, and amino acid metabolism, with some involved in the biosynthesis of secondary metabolites.

### Antimicrobial purification and characterization

**Figure 3.**
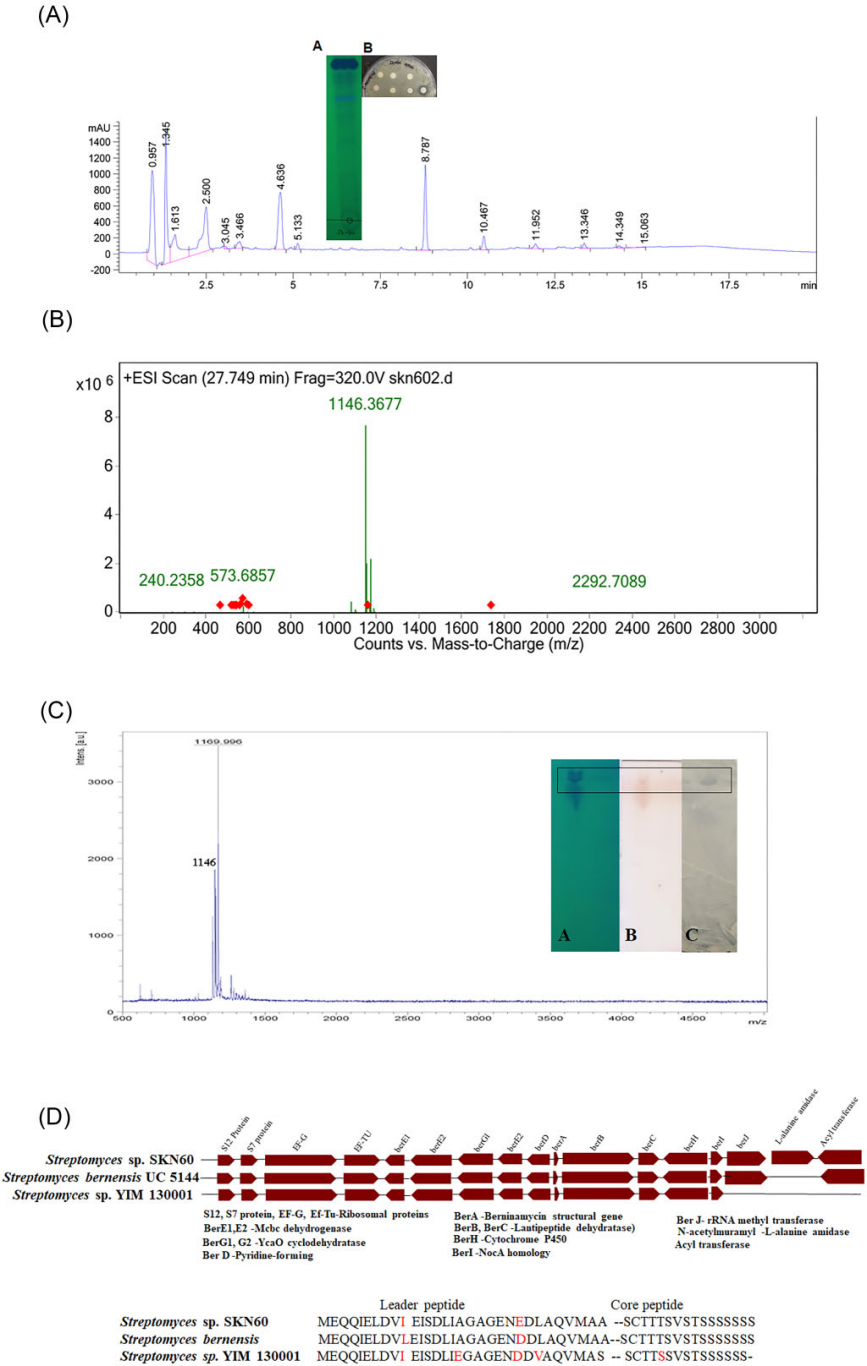
Purification and characterization of antimicrobial substance produced by SKN60^T^. **(A)** HPLC analysis of crude extract (inset TLC showing multiple bands under UV light 254 nm and antimicrobial activity of HPLC fractions at different retention time). **(B)** Q-TOF spectrum of the peak showing representative precursor ions (m/z, 1146.3 [M+H], 573.6 [M+2H]^2+^). **(C)** MALDI result of purified AMP showed intact peak of 1169 [M+Na] as sodium adduct (inset TLC showing a single band in UV light and stained with ninhydrin. The bioautography analysis showed inhibition of *S. aureus* MTCC 1430 using agar overlay assay). **(D)** Organization of genes in BGC and comparison with *S. bernensis* UC5144 and *Streptomyces* sp. YIM130001 with their peptide sequence precursors encoded by the structural gene. Arrow in between the peptide sequence indicated the demarcation between leader and core amino acid sequences.

The growth of *Streptomyces* sp. strain SKN60^T^ was analyzed up to 8 days in *Streptomyces* broth at 30°C and shaking condition with biomass determination at 24 h regular time intervals. Simultaneously, the CFB obtained was used to determine antimicrobial substance production by testing inhibitory activity on indicator strain *S. aureus* MTCC 1430. Cell biomass production of strain SKN60^T^ displayed a steady increase in cell biomass up to 5 days of incubation and thereafter decrease in the biomass was observed. An agar well diffusion assay showed inhibition of *S. aureus* MTCC 1430 from day 3 onwards with maximum zone of inhibition (13 mm) at day 5. Among the media used for antimicrobial substance production, *Streptomyces* broth yielded good growth of strain SKN60^T^ and also displayed maximum inhibition zone against *S. aureus* MTCC 1430. Similar biomass yield and activity were also observed with NB medium. The CFB obtained inhibited Gram-positive bacterial indicator strains but did not inhibit Gram-negative test strains. The antimicrobial substance was extracted using ethyl acetate solvent and used as a crude extract for further purification. HPLC analysis of crude extract showed multiple peaks (Fig. 3A) and TLC showed multiple bands (inset A Fig. 3A), but only the HPLC peak eluted at 8.7 min retention time showed promising inhibitory activity against *S. aureus* MTCC 1430 (inset B Fig. 3A). The mass of the peptide was determined by LC-MS (Q-ToF), which showed a predominant peak with mass m/z 1146.3 Da (Fig. 3B). A minor peak observed with mass m/z 573 Da shows presence of precursor ion corresponding to M+H^2+^. The fraction showing activity was repeatedly injected on HPLC and collected manually. Mass spectrum (MALDI) analysis of collected fragment showed a predominant peak with mass signal m/z 1169 Da (Fig. 3C) and another peak at 1146 Da (M+H^+^). The 23 Da difference observed between both peaks indicated the formation of sodium adduct. Analysis of the sample on TLC showed a distinct band under UV light (inset A Fig. 3C) and stained with ninhydrin (inset B Fig. 3C). It also displayed activity against *S. aureus* MTCC 1430 in bioautography assay (inset C Fig. 3C).

### BGC analysis

The bioinformatic analysis of *Streptomyces* sp. strain SKN60^T^ genome sequence was carried out to determine genes encoding secondary metabolites using antiSMASH 7.0 software. Results revealed that 23 putative BGCs encode various secondary metabolite synthesis with different similarity index (Table S2, Supporting Information). One of the BGCs showed the presence of genes involved in the synthesis of a RiPP (ribosomally synthesized post-translationally modified peptide). The predicted cluster contained 17 ORFs (spans ~30 kb) encoding all genes required for thiopeptide biosynthesis including enzymes involved in post-translational modifications. Bioinformatic analysis of the BGC revealed 94% identity to berninamycin, however, translated sequence of thiopeptide encoding *berA* gene showed minor difference in the amino acid composition of the leader peptide (Fig. 3D). The structural gene encoding thiopeptide (*berA*) contained a total of 74 amino acids. The core peptide had 16 amino acids with Serine (Ser) residue at the 10th position and was identical to berninamycin amino acid composition (Fig. 3D). The remaining clusters comprised the hallmark of thiopeptide synthesis, such as BerB-D encoding lantibiotic dehydratase N- and C-terminus, pyridine forming genes, respectively. BerE1 and E2 were observed for installing the lantibiotic dehydrogenase. BerG1 and G2 encompass YcaO-type cyclodehydratases and docking scaffold that control the synthesis of thiazole, oxazole, and methyloxazole. Additionally, BerI encodes for C-terminal amide-forming enzymes. Nevertheless, an additional gene was observed in the biosynthetic cluster of strain SKN60^T^ encoding l-alanyl peptidase, which is known to carry out cleavage of the propeptide during the maturation of active peptide.

### Antimicrobial activity

**Figure 4.**
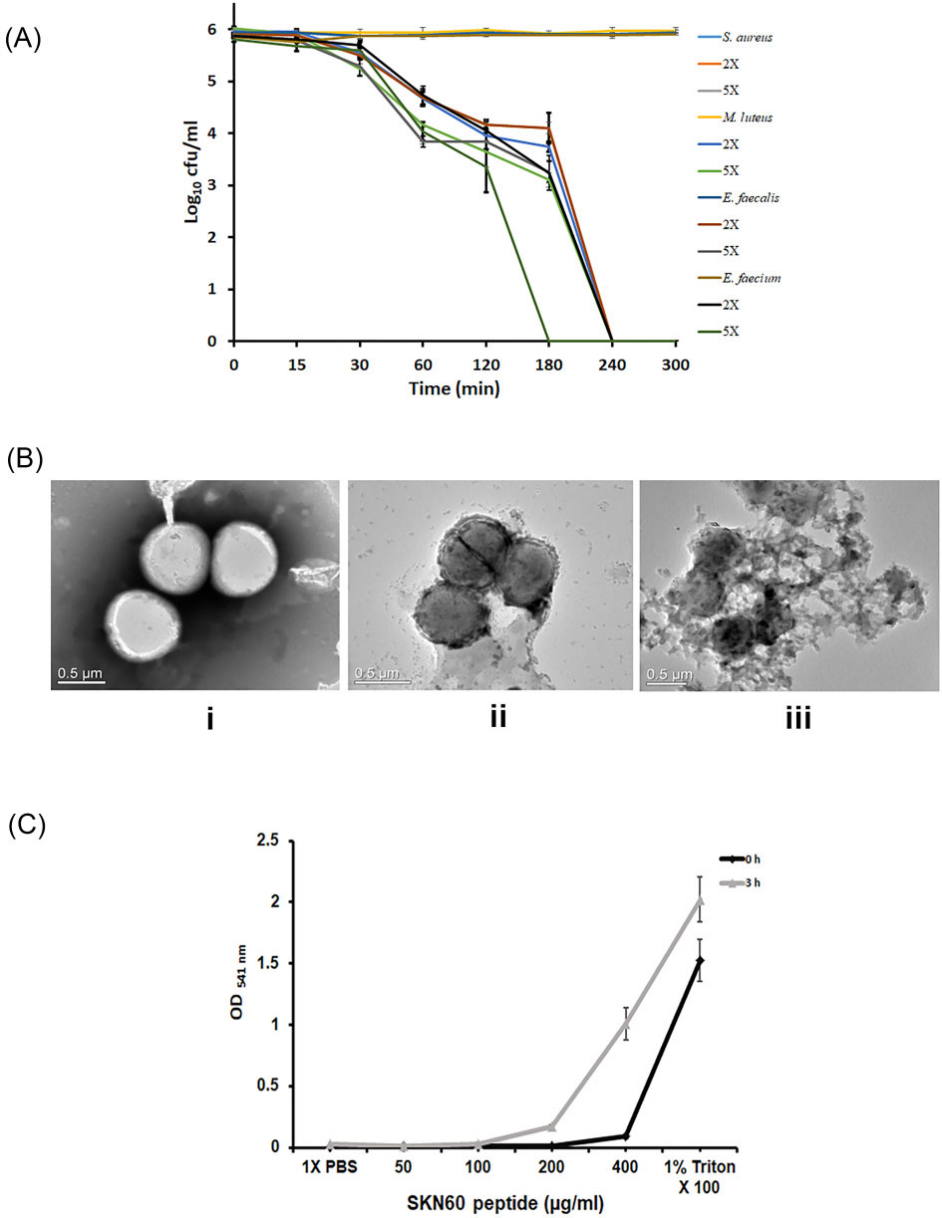
Determination of antibacterial and haemolytic activities. **(A)** Time–kill assay against Gram-positive bacterial strains. **(B)** TEM visualization of membrane disintegration by antimicrobial substance: (i) untreated cells of *S. aureus* MTCC 1430, (ii) *S. aureus* MTCC 1430 cells treated with 2X MIC of SKN60^T^ peptide, and (iii) treated with 5X MIC of SKN60^T^ peptide. **(C)** Determination of haemolysis by SKN60^T^ peptide showing lysis of RBCs at higher concentration upon 3 h of incubation.

*In vitro* antimicrobial studies showed that the antimicrobial peptide from strain SKN60^T^ inhibited the growth of Gram-positive bacteria. Details of MIC evaluation results observed for various strains are provided in Table 1. MIC of antimicrobial peptide corresponded to 60, 70, and 90 µg ml^−1^ against *Enterococcus faecium* MTCC 789, *Enterococcus faecalis* MTCC 439, and *S. aureus* MTCC 1430, respectively. Further, MIC determination of SKN60^T^ peptide against various species of the genus *Bacillus* ranges from 70 to 90 µg ml^−1^. Nevertheless, no effect was detected against Gram-negative indicator strains including *P. aeruginosa* MTCC 1934 and *E. coli* MTCC 1610. Results of killing kinetics of the peptide showed a reduction of growth of test strains as 2-log kill was observed within 1 h of incubation with peptide (5X MIC) for all strains (Fig. 4A). Amongst test strains, *E. faceium* MTCC 789 showed complete killing within 3 h of incubation with a peptide concentration of 5X MIC. Overall, within 4 h of incubation the growth of all strains was inhibited. Electron microscopic observation of untreated cells (control) showed intact cells with a smooth surface. TEM analysis showed the disintegration of cell membrane upon exposure to the peptide (Fig. 4B). It also showed complete lysis with the accumulation of cell lysate debris in surroundings at higher peptide concentration.

**Table 1.** MIC values of antimicrobial peptide from SKN60^T^ against various Gram-positive bacterial test strains.

Test strains	MIC (µg ml^−1^)
*M. luteus* MTCC 106	50
*E. faecium* MTCC 789	60
*L. monocytogenes* MTCC 839	60
*E. faecalis* MTCC 439	70
*Streptococcus oralis* MTCC 2696	70
*S. aureus* MTCC 1430	90
*B. subtilis* MTCC 121	70
*B. megaterium* MTCC 428	70
*B. coagulans* MTCC 492	70
*B. tequilensis* MTCC 619	70
*B. circulans* MTCC 490	70
*B. wiedmannii* MTCC 453	80
*B. halodurans* MTCC 865	80
*B. firmus* MTCC 488	90
*B. fastidous* MTCC 1308	90

### Haemolysis

The haemolytic activity of the compound was confirmed by testing its effect on New Zealand white rabbit RBCs. It was observed that 400 µg ml^−1^ concentration of peptide showed about 5% of haemolysis with respect to the positive control (1% Triton X100) observed at 0 h time point. However, haemolysis was increased to 66% with 400 µg ml^−1^ concentration upon 3 h of incubation (Fig. 4C).

## Discussion

Members of the genus *Streptomyces* are ubiquitous and received much attention due to their ability to produce secondary metabolites with extensive medicinal value. However, most of the *Streptomyces* strains were not explored for antimicrobial metabolites, as revealed by genome sequences. Further, research is reviving for the undiscovered bioactive molecules through genome mining of available sequences (Smanski et al. 2016). Therefore, aiming to isolate bacterial strains producing such antimicrobial compound, we have plated fertile subsurface soil sample on actinomycetes medium. One of the bioactive producing isolates, strain SKN60^T^ with actinomycetes like colony features was studied in detail. The phenotypic features including colony morphology, fatty acids composition, polar lipid profile, and G+C mol% contents align with the properties described for the genus *Streptomyces*. The 16S rRNA gene sequence analysis of SKN60^T^ showed closest sequence identity (99.31%) to *S. laurentii* ATCC 31255^T^. Subsequent genome sequence analyses of strain SKN60^T^ confirmed the maximum ANI (88.45%) and dDDH (33.7%), respectively, with *S. roseiocoloratus* TRM44457^T^. However, these values are much lower than the threshold values required for species delineation (Rossello-Mora and Aman 2001), and thus strain SKN60^T^ represents a novel species of the genus *Streptomyces*.

It is reported that *Streptomyces* strains are known to contain an enormous repository of BGCs encoding secondary metabolites with a promising ability for the secretion of novel antimicrobials (Nandhini et al. 2018, Pacios-Michelena et al. 2021, Alam et al. 2022). Accordingly, strain SKN60^T^ identified as a member of the genus *Streptomyces* and its genome analysis by antiSMASH showed the potential to secrete bioactive compounds. Detailed analysis showed the presence of a RiPP cluster showing 94% sequence identity with thiopeptide berninamycin. The unique feature observed in the BGC of berninamycin is the presence of genes encoding cyclodehydratase and dehydrogenase involved in post-translational modifications, which are observed in strain SKN60^T^. Further, considering the enzyme activity, the presence of cysteine, serine and threonine residues at a conserved region in propeptide clearly suggests it to be a substrate for the enzymes involved in post-translational modifications. Schneider et al. (2018) observed these modifications are essentially required in synthesis of geninthiocin, a thiopeptide antibiotic. These modifications were in alignment with observed mass of 1146 Da (M+H^+^) for recombinant version of berninamycin (Malcolmson et al. 2013). BGC of berninamycin showed BerH gene belongs to P450 hydroxylase family, which is mostly involved in the hydroxylation of valine. These genes are responsible for hydroxylation at the tailoring stage after the formation of main scaffold (Malcolmson et al. 2013, Schneider et al. 2018). A previous study demonstrated the involvement of gene BerI in post-translational modifications that acts on a macrocyclic substrate to yield C-terminal amide and generates active berninamycin (Malcolmson et al. 2013). It is pertinent to mention that the presence of l-alanyl peptidase only observed in berninamycin biosynthetic cluster of strain SKN60^T^ is typically known to carry out the cleavage of the peptide at N-terminal alanine residue perhaps to yield active peptide. The subtle difference observed in the leader peptide region of *berA* gene sequence indicates the microevolution of BGCs with subsequent effect on secretion levels (Smanski et al. 2016).

Thiopeptides or thiazolyl peptides reported mainly from genus *Streptomyces* are well-known antimicrobial compounds with complex structural configurations. The research in this class of antibiotics has gained significant attention since it showed promising antimicrobial activity against drug-resistant pathogens, including penicillin-resistant *Streptococcus pneumoniae*, methicillin-resistant *S. aureus*, and vancomycin-resistant enterococci (Schneider O et al. 2018, Shen et al. 2019). Studies demonstrated that berninamycin and its variants produced by *Streptomyces* species showed antibacterial activity against Gram-positive bacteria (Bagley et al. 2005, Schneider et al. 2018, De BC et al. 2021). Strain SKN60^T^ was cultured on different carbon and nitrogen sources to investigate the possibility of variation in antimicrobial compound production. The cell-free broth of strain SKN60^T^ showed antibacterial activity against indicator strains of Gram-positive bacteria like *S. aureus* MTCC 1430, *E. faecalis* MTCC 439, and *E. faecium* MTCC 789. Notably, the antibacterial activity was also in alignment with berninamycin. However, thiopeptides were also known to have antifungal, antiplasmodial, and anticancer activities (Aminake et al. 2011, Ovchinnikov et al. 2021). In contrast, SKN60^T^ peptide did not inhibit the growth of yeast and fungi, indicating its potential to differentiate between prokaryotic and eukaryotic cell membranes.

Thiopeptide antibiotics like nosiheptide thiocillin, thiostrepton, and geninthiocin were known to block protein synthesis resulting in the death of cells (Mikolajka et al. 2011, Cundliffe and Thompson 1981). Similarly, thiopeptide from strain SKN60^T^ in this study caused cell death and TEM analysis showed disruption of cell integrity of *S. aureus* MTCC 1430. Although antimicrobial peptides have gained significant attention over the past two decades, but problems such as haemolysis, cytotoxicity and poor efficacy under *in vivo* conditions have limited their clinical value. Similarly, SKN60^T^ peptide showed haemolysis of RBCs at a higher concentration that increased with prolonged incubation. The recent success in developing analogues of thiopeptide with remarkable pharmaceutical applications may pave the way for developing drugs targeting antimicrobial-resistant pathogens (Just-Baringo et al. 2014). Although berninamycin was reported from *S. bernensis*, such strain is not available in public domain and it is not validated as a species of the genus *Streptomyces*. This is first study to report the production of berninamycin by an isolate, which is identified as a novel species of the genus *Streptomyces*. It is important to note that such antimicrobial producing strains are not available in public culture collection, but their genome sequences are deposited in databases. Thus, it hinders the comparison studies, a complete understanding of the antimicrobial production and strain improvement to increase the yield.

Taken together, our results indicate that *Streptomyces* sp. strain SKN60^T^ is a novel species isolated from agricultural soil with low sequence identity with all type strains of the genus *Streptomyces*. In this study, we have reported the production of thiopeptide berninamyin from a type strain as confirmed by mass and sequence analyses. This bioactive molecule showed inhibitory activity against Gram-positive bacteria. Nevertheless, further research must be carried out to elucidate the molecular mechanisms underlying the synergistic effects and the exact mechanism behind the bactericidal effect. Such information/knowledge will help design more efficient and safer drugs before the clinical trials.

## Description of novel species

### *Streptomyces terrae* (terʹrae. L. Gen.n. *Terrae* pertaining to soil, referring to isolation source)

Strain SKN60^T^ is an aerobic, Gram-positive, nonmotile filamentous actinobacteria. The substrate mycelium was well-grown and varied from off-white to brownish on different media. Growth is observed from 20 to 40ºC and pH ranging 4–10. It tolerates up to 8% NaCl and showed a positive reaction for nitrate reduction. Utilization of sugars like arabinose, dulcitol, sucrose, galactose, inositol, lactose, mannose, melibiose, mannitol, raffinose, rhamnose, salicin, sucrose, and trehalose is positive. Major cellular fatty acids are iso-C_15:0_, anteiso-C_15:0_, iso-C_16:0_, and iso-C_17:0_ 3-OH. Predominant polar lipids are DPG, PE, and PC. The cell wall peptidoglycan contains meso-DAP. The G+C content is 72.3%. The type strain SKN60^T^ (MTCC 13163^T^, JCM 3577^T^) is isolated from an arable subsurface soil sample.

## Supplementary Material

xtad014_Supplemental_Files

## Data Availability

The GenBank accession number for the 16S rRNA gene sequence is MZ642351 and the genomic assembly accession for the strain SKN60^T^ is JAHTMW000000000. Strain SKN60^T^ deposited with accession numbers at Microbial Type Culture Collection and Gene bank MTCC 13163^T^ and Japan Collection of Microorganisms JCM 3577^T^.
