# Supplementary material for: An antimicrobial thiopeptide producing novel actinomycetes Streptomyces terrae sp. nov., isolated from subsurface soil of arable land
Source: FEMS Microbes. 2023 Aug 10;4:xtad014. doi: 10.1093/femsmc/xtad014 (PMC10495126; doi:10.1093/femsmc/xtad014)
Supplement: xtad014_Supplemental_Files [file xtad014_supplemental_files.zip › Supplementary Information Strain SKN60.pdf]

An antimicrobial thiopeptide producing novel actinomycetes *Streptomyces terrae* sp. nov., isolated from subsurface soil of arable land

Stanzin Choksket, Mahaldeep Kaur, Anil Kumar Pinnaka, Suresh Korpole*

CSIR-Institute of Microbial Technology, Sector 39A, Chandigarh, India.

*Corresponding author: Suresh Korpole, CSIR-Institute of Microbial Technology, Chandigarh-160036, India, email: suresh@imtech.res.in

Supplementary Tables:

Supplementary Table S1: Differentiating characteristics of strain SKN60^T and it's closet phylogenetic neighbors of genus *Streptomyces.* + : posiive; - : negative; NA: Not available

**Characteristic features**	**SKN60^T**	***S. roseicoloratus* TRM 44457^T**	***S. laurentii* ATCC 31255^T**
Growth	Abundant	Good	Good
Aerial Mycelium	Creamish	Light pink	Creamish
Soluble pigment	Brownish	-	-
Sporulation	white	White pink	Red
Oxidase	+	-	NA
Catalase	-	-	NA
Temperature (°C)	20-40	8-45	28
NaCl (%)	2-8	0-10	NA
pH tolerance	4-10	4-11	5-8
Starch hydrolysis	+	+	+
Gelatin hydrolysis	-	+	+
Skim milk/ Caesin hydrolysis	+	+	NA
Arginine dihydrolase	-	NA	+
Urease	-	-	+
Citrate Utilization	-	+	+
Indole test	-	NA	-

Voges Proskauer test (VP)	-	NA	-
H_2S production	+	-	NA
Nitrate reduction	+	+	NA
Galactose	+	+	+
Arabinose	+	+	-
Lactose	+	+	+
Trehalose	+	NA	-
Sorbitol	+	NA	-
Melibiose	+	NA	+
Mannitol	+	+	-
Raffinose	+	+	-
Sucrose	+	+	+
Fucose	-	+	-
Fructose	-	+	-
Rhamnose	+	+	-
Xylose	+	+	+
Inositol	+	+	-
Polar lipids	DPG, PE, PG	DPG, PE, PC and PI	NA
GC mol%	72.3	72.6	72.3

Supplementary Table S2: Different types of clusters identified in strain SKN60^T showing higher than 50% similarity with biosynthetic gene clusters involved in secondary metabolites synthesis.

**S No**	**Most similar known cluster**	**Type**	**Region**	**Total Nucleotide(bp)**	**Cluster class**	**Identity** (%)
1	Ectoine	Ectoine	18.1	10239	Osmolyte	100
2	Desferrioxamin B		1.2	11784	Siderophore	100
3	Berninamycin K/Berninamycin J/Berninamycin A/Berninamycin B	Ripp	3.2	30806	Thiopeptide	94
4	Flaviolin	Polyketide	7.1	50643	Terpene, T3PKS	75
5	Hopene	Terpene	5.4	26120	Terpenoids	69
6	Flaviolin/1,3,6,8-Tetrahydroxynaphthalene	Polyketide	4.1	41133	T3PKS	66
7	Pyridomycin	NRP+Polyketide	9.3	111765	Phophonate, T1PKS, NRPS	65

Supplementary Figures:

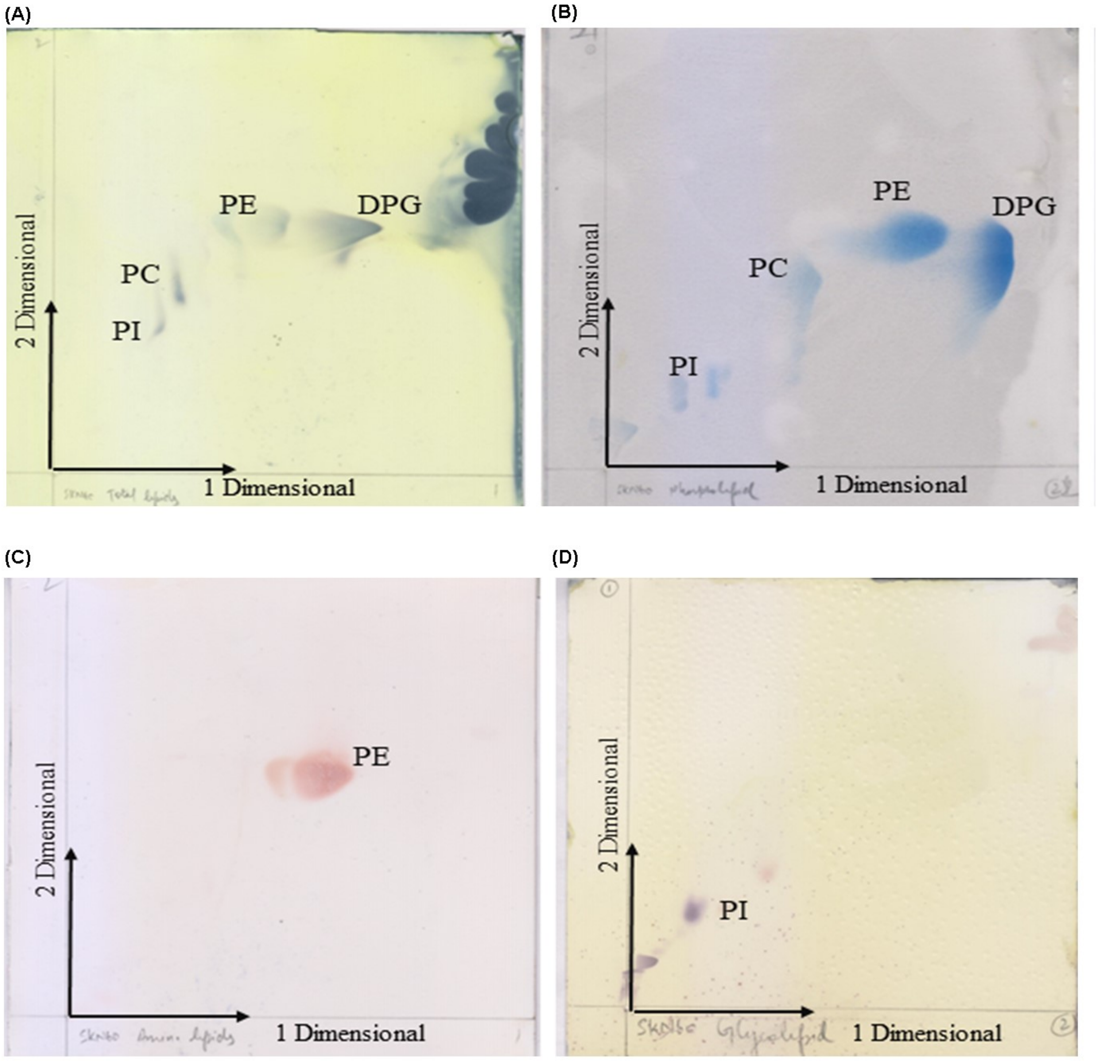

Supplementary Figure S1: Two-dimensional thin-layer chromatography (TLC) analysis of polar lipids extracted from strain SKN60^{T}. (A) TLC plate sprayed with molybdotophosphoric acid for the detection of total lipids profile. (B) TLC plate sprayed with molybdenum blue for phospholipids detection. (C) TLC plate sprayed with ninhydrin for amino lipids detection. (D) TLC plate sprayed with α-naphthol for the detection of glycolipids. DPG, diphosphatidylglycerol; PE, phosphatidylethanolamine; PC, phosphatidylcholine and PI, phosphotidylinositol.

(A)

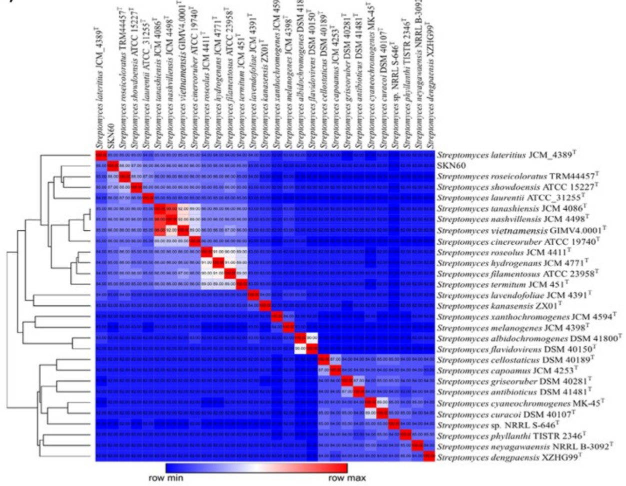

(B)

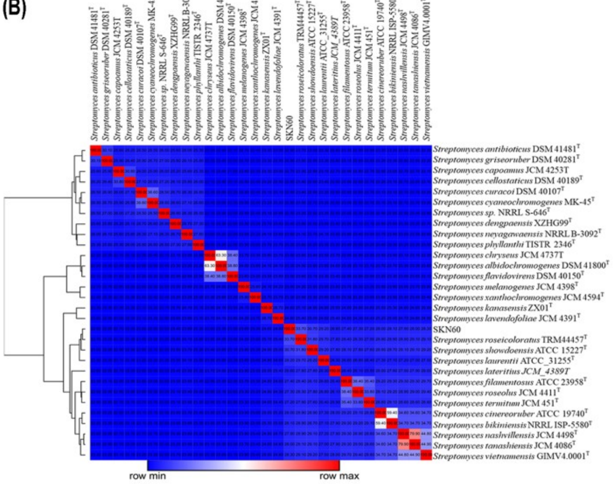

(C)

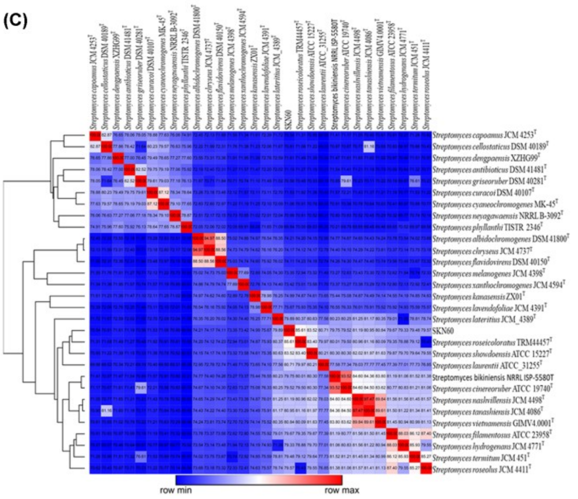

Supplementary Figure S2: Construction of matrix based heatmaps showing similarity values acquired amongst different closest representative type strains of SKN60^{T} (A) Average Nucleotide Identity (ANI), (B) *in silico* DNA-DNA hybridization (dDDH) and (C) Average amino acid identity (AAI). Red and blue colours indicate maximum and minimum values, respectively.

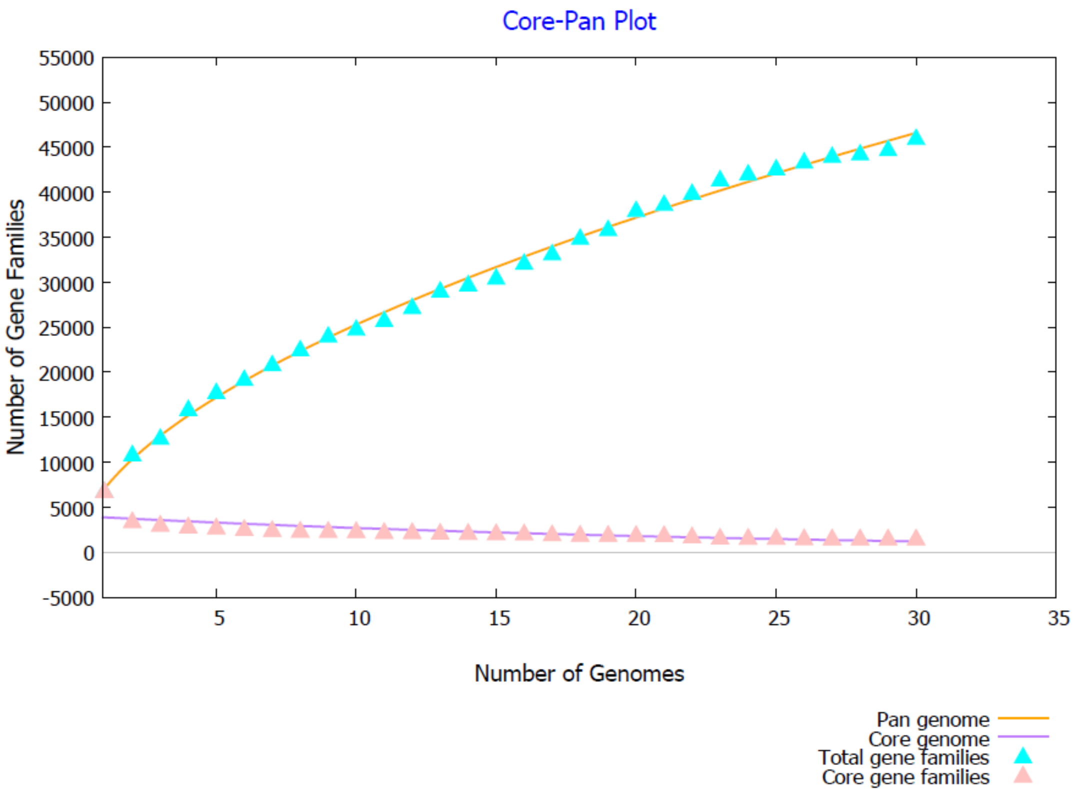

Supplementary Figure S3: Core-Pan genome plot study of SKN60^{T} and its phylogenetic closest representative type strains of the genus *Streptomyces*. The X-axis in the graph denotes the number of closest strains and Y-axis indicates the number of genes expressed in each strain. The orange line in the chart indicates the gradual pan-genome expansion with the addition of genomes. The purple line shows the core-genome that remained conserved for the mentioned genomes during evolution. The addition of new gene families (marked as a blue triangle) suggests an open-type pan-genome, while core gene families (marked as a pink triangle) are shown as conserved among all the genomes. With the addition of the new genomes, there observed a gradual decrease in the core-genome and an increase in the number of new gene families in pan-genome.
