# Supplementary material for: An antimicrobial thiopeptide producing novel actinomycetes Streptomyces terrae sp. nov., isolated from subsurface soil of arable land
Source: FEMS Microbes. 2023 Aug 10;4:xtad014. doi: 10.1093/femsmc/xtad014 (PMC10495126; doi:10.1093/femsmc/xtad014)
Supplement: xtad014_Supplemental_Files [file xtad014_supplemental_files.zip › Supplementary_Data_-_FEMSMC-2023-009_reviews_and_author_response.docx]

**Reviews of manuscript FEMSMC-2023-009**

**Reviewer 1: XueWei Xu, Second Institute of Oceanography, Laboratory of Marine Ecosystem and Biogeochemistry**

**Recommendation: Minor Revision**

Authors isolated a species of genus Streptomyces, and strain SKN60T was systematically identified as a novel species in this genus using polyphasic taxonomic approach. The antimicrobial products of this species were predicted through bioinformatics methods, the antibacterial compounds were extracted and their biological activity were verified through experiments. At the same time, strain SKN60T was the only strain containing berninamycin BGC as no other phylogenetic relative had it. This article is well written with adequate data, but there are still some shortcomings in the identification of new species. Given the high 16S rRNA similarity between strain SKN60T and reference strains, it is recommended to stress on the description of the differences between strain SKN60T and reference strains in the morphological and physiological results section. Besides, the tense of sentences describing experimental results and conclusions should be consistent.

1. Line 24, “SKN60” in ‘Abstract’ should be changed to “SKN60T”, and it is suggested to make the same modifications elsewhere.

2. It is recommended to briefly introduce the shape, Gram staining, motility, and whether the strain SKN60T can grow without oxygen in ‘Abstract’.

3. The abstract should include information on the highest 16S rRNA similarity, AAI, ANI, dDDH, etc. between strain SKN60T and the same strain.

4. The Phenotypic Characterization in Materials and Methods should include morphological, physiological, and chemical characteristics. This paragraph mainly discusses the isolation, cultivation, morphological and physiological characteristics of strains, which is inconsistent with the title of this paragraph. At the same time, the introduction in this paragraph is too brief and should provide a detailed and comprehensive description of experimental materials and methods. It is recommended to separate the isolation and cultivation of the strain into one paragraph, as well as the morphological and physiological characteristics.

5. It is recommended to place the “16S rRNA gene sequence analysis” in Materials and Methods after the " Chemotaxonomic characterization".

6. Line 136, “Vernikos et. (2015)” is an inappropriate expression, which should be replaced by “Vernikos et al. (2015)”.

7. Line 177, "is" redundant.

8. Line 217-240, given the high 16S rRNA gene similarity between strain SKN60T and reference strains, the physiological differences between strain SKN60T and reference strains should be presented in a table format.

9. “SKN60” in the phylogenetic tree in Figure 1 should be replaced by the scientific name of the strain and shown in bold. The format of the strain accession numbers in the figure is not standardized and should be shown in (). The outgroup lacks a scientific strain name.

10. Line 245, the expression “NBRC13194T” is not standardized, and there should be a space between the abbreviation of the microbiological culture collection centre and the preservation number. It is also recommended to make the same changes in other areas.

11. The identification results of Menaquinone were not presented in the article.

12. Only the “antiSMASH 3.0 database tool” is introduced in the Materials and Methods, and the “antiSMASH 7.0 software” mentioned in line 323 is not introduced in the Materials and Methods.

13. Line 439, “terrae” requires italics.

14. Line 442, “upto” should be changed to “up to”.

15. Line 445, “iso C15:0”should be changed to “iso-C15:0”, “iso C16:0”should be changed to “iso-C16:0”, “C17:0 3OH” should be changed to “C17:0 3-OH”. It is also recommended to make the same changes in other areas.

16. Line 447-448, “The type strain is SKN60T (MTCC 13163T, JCM 3577T) isolated from” should be changed to “The type strain SKN60T (MTCC 13163T, JCM 3577T) is isolated from”.

**Reviewer 2: Roshan Kumar, Magadh University, Post-Graduate Department of Zoology**

**Recommendation: Major Revision**

The manuscript entitled “An antimicrobial thiopeptide producing novel actinomycetes Streptomyces terrae sp. nov., isolated from subsurface soil of arable land” by Choksket et al, isolated and identified a novel bacterial strain from genus Streptococccus, showed its potential to produce the antimicrobial thiopeptide. Altogether, the study has scientific merit, but I am not sure whether its good fit for FEMS Microbe journal.

I have some concerns pertaining to the content of this article mentioned as follows:

1. The introduction (lines 77-80) states that the objective was to identify the microbe responsible for producing thiopeptides, while in the results section (line 217), the aim was to screen the rhizosphere soil sample for microorganisms exhibiting antimicrobial activity. However, the methodology lacks a section that specifies whether the isolation of isolates was based on thiopeptide-specific selection or other antimicrobial criteria. This deficiency highlights a major flaw in the experimental design or the hypothesis derived from the results. Therefore, it is necessary to address this issue to ensure the accuracy and reliability of the study.

2. It is suggested to add a few lines outlining the isolation protocol in line 83.

3. Please add a statement in line 98 describing the method used to generate the consensus 16S rRNA gene sequence from the Sanger dataset.

4. Please provide more details on how Quast was used to identify the conserved genes as I am not aware it does and add a reference for BUSCO.

5. In line 232, please include the range, optimum temperature, pH, and NaCl tolerance values.

6. It is recommended to use the 16S rRNA gene from the genome, rather than the one generated from the Sanger sequencing method, to construct the phylogenetic tree. Sanger sequencing should only be used for isolate identification.

7. In line 252, please state the genome size in Mb and include the bp in the N50 contig size value.

8. Line 255 should be removed as it is part of the method, not the result.

9. Please revalidate the ANI and AAI results based on comment 6.

10. Line 285 needs to be clarified.

11. In lines 289 and 291, the pangenome and core genome are two separate concepts and should not be confused. The paragraph should be modified to avoid unnecessary confusion for readers.

12. What does "ca" mean in line 327?

13. In line 359, what does "0 h" refer to? Is it a zero time point?

14. The manuscript appears to be written in a format suitable for classification and taxonomical characterization for a specific journal type. However, it may need to be reformatted for FEMS Microbe journal or audience.

**Author's Response to Decision Letter for (FEMSMC-2023-009)**

Responses to the queries raised by the reviewers:

Manuscript: FEMSMC-2023-009-An antimicrobial thiopeptide producing novel actinomycetes *Streptomyces terra*e sp. nov., isolated from subsurface soil of arable land

**Reviewer: 1**

Comments to the Author

Authors isolated a species of genus Streptomyces, and strain SKN60T was systematically identified as a novel species in this genus using polyphasic taxonomic approach. The antimicrobial products of this species were predicted through bioinformatics methods, the antibacterial compounds were extracted and their biological activity were verified through experiments. At the same time, strain SKN60T was the only strain containing berninamycin BGC as no other phylogenetic relative had it. This article is well written with adequate data, but there are still some shortcomings in the identification of new species. Given the high 16S rRNA similarity between strain SKN60T and reference strains, it is recommended to stress on the description of the differences between strain SKN60T and reference strains in the morphological and physiological results section. Besides, the tense of sentences describing experimental results and conclusions should be consistent.

- We thank the reviewer for the positive feedback and all the suggestions to improve the quality of our manuscript.

1. Line 24, "SKN60" in 'Abstract' should be changed to "SKN60T", and it is suggested to make the same modifications elsewhere.

- As suggested by the reviewer, we have modified "SKN60" to "SKN60^T^" in the abstract (Line 31) as well as throughout the manuscript.

2. It is recommended to briefly introduce the shape, Gram staining, motility, and whether the strain SKN60T can grow without oxygen in 'Abstract'.

- As suggested by the reviewer, we have briefly mentioned the phenotypic features of strain SKN60^T^. “An antimicrobial producing Gram-positive, aerobic, non-motile and filamentous actinobacterial strain SKN60^T^ was isolated from soil.” (Line 30).

3. The abstract should include information on the highest 16S rRNA similarity, AAI, ANI, dDDH, etc., between strain SKN60T and the same strain.

- We have mentioned the suggested information as: “The isolate exhibited 99.3 and 99.0% identity with *Streptomyces* *laurentii* ATCC 31255^T^ and *S. roseicoloratus* TRM 44457^T^, respectively in 16S rRNA gene sequence analysis*.* However, the genome sequence displayed maximum ANI (88.45%) and AAI (85.61%) with *S. roseicoloratus* TRM 44457^T^. Similarly, the dDDH showed 33.7% identity with *S. roseicoloratus* TRM 44457^T^. (Line 31-35).

4. The Phenotypic Characterization in Materials and Methods should include morphological, physiological, and chemical characteristics. This paragraph mainly discusses the isolation, cultivation, morphological and physiological characteristics of strains, which is inconsistent with the title of this paragraph. At the same time, the introduction in this paragraph is too brief and should provide a detailed and comprehensive description of experimental materials and methods. It is recommended to separate the isolation and cultivation of the strain into one paragraph, as well as the morphological and physiological characteristics.

- As suggested by the reviewer material and methods section is included with “Soil sample collection and isolation of actinomycetes (new addition), Morphological and physiological characterization and Chemotaxonomic characterization” in the revised manuscript
- **Soil sample collection and isolation of actinomycetes:** Soil sample was collected from subsurface (20 cm depth) environment of an arable land in Chandigarh and processed immediately to screen microorganisms exhibiting antimicrobial activity. Briefly, 1g of soil was serially diluted in 10 ml of sterile saline and 100 µl aliquots of each dilution (10^-2^ to 10^-4^) were inoculated on starch casein agar (Himedia, India) plates. The plates were incubated at 30ºC for 3-4 days and observed after every 24 h time interval for antimicrobial producing strains. Colonies showing inhibitory zone in their periphery were picked up and streaked on streptomyces agar (Himedia, India) for further purification, and stored as glycerol stocks in -70 ºC deep freezer until further use. A strain designated as SKN60 was selected for detailed studies. (Line 94-101)

5. It is recommended to place the "16S rRNA gene sequence analysis" in Materials and Methods after the "Chemotaxonomic characterization".

- We have incorporated the suggestion in the revised manuscript
- **Chemotaxonomic characterization**

Lyophilized cell biomass of strain SKN60^T^ was used for different chemotaxonomic analysis. The fatty acid methyl esters (FAMES) were analysed by Sherlock Microbial Identification System (MIDI) using version 6.1 (Sasser 1990). Total cellular polar lipids, menaquinone and peptidoglycan analysis were performed by thin layer chromatography (TLC) analyses (Minnikin et al. 1984, Schumann, 2011). The extracted polar lipids were analysed using two-dimensional TLC. Different staining reagents, including molybdatophosphoric acid, ninhydrin, molybdenum blue and α-naphthol reagents, were employed to detect total lipids, aminolipids, phospholipids and glycolipids, respectively. (Line 115-121)

- **16S rRNA gene sequence analysis**

The genomic DNA of SKN60 was extracted using Zymo DNA isolation kit and used for 16S rRNA gene amplification. Briefly, 16S rRNA gene was amplified using the primers 27F and 1492R and 5x firepol master mix (SolisBioDyne, Estonia) in a 50µl reaction volume. The amplified product was run using 1% agarose gel and purified using HiYield™ Gel/PCR DNA mini kit (Real Biotech Corporation). The purified amplicon was sequenced using three internal primers including 27F, 786F and 1492R (10µl reaction volume) and the sequencing was performed on an ABI Prism 3700 automatic DNA sequencer (Applied Biosystems). The nucleotide sequence files (.ab1) were analysed using FinchTV version 1.4.0 software and assembled manually. The consensus sequence was used for BLAST sequence similarity search on EzTaxon server (Chun et al. 2007). All closely related sequences were retrieved in fasta format and aligned with SKN60^T^ sequence using CLUSTAL_W (Thompson et al. 1994). The phylogenetic tree was constructed by neighbour-joining algorithm using MEGA software version 7.0 (Tamura et al., 2011). Kimura two-parameter was used to calculate evolutionary distances and the tree topologies were evaluated by bootstrap analyses with 1000 replications. (Line 123-136)

6. Line 136, "Vernikos et. (2015)" is an inappropriate expression, which should be replaced by "Vernikos et al. (2015)".

- Typographic mistake is corrected as “Vernikos et al. (2015)” (Line 163) as suggested by the reviewer.

7. Line 177, "is" redundant.

- Sentence corrected as “The purified fraction was collected manually and used for further studies” (Line 203-204)

8. Line 217-240, given the high 16S rRNA gene similarity between strain SKN60T and reference strains, the physiological differences between strain SKN60T and reference strains should be presented in a table format.

- As suggested, we have provided the physiological differences between strain SKN60^T^ and reference strains in a Supplementary Table S1 in the revised version of the manuscript.

9. "SKN60" in the phylogenetic tree in Figure 1 should be replaced by the scientific name of the strain and shown in bold. The format of the strain accession numbers in the figure is not standardized and should be shown in (). The outgroup lacks a scientific strain name.

- We thank the reviewer for pointing this out. We have revised Figure 1 as suggested. In addition, we have rectified "SKN60" mentioned in Figure 1c with the standard format (the scientific name of the strain) as "*Streptomyces* species strain SKN60".

10. Line 245, the expression "NBRC13194T" is not standardized, and there should be a space between the abbreviation of the microbiological culture collection center and the preservation number. It is also recommended to make the same changes in other areas.

- We have made the changes as suggested by the reviewer in the revised manuscript as “The maximum sequence identity was observed with type strains of *S. laurentii* ATCC 31255^T^ (99.31%), *S. roseicoloratus* TRM 44457^T^ (99.03%), *S. showdoensis* NBRC 13417^T^ (98.47%) and *S. vietnamensis* GIMV4.0001^T^ (98.31%). The phylogenetic tree constructed using neighbour-joining phylogenetic analysis showed strain SKN60^T^ forming a cluster with closest phylogenetic relatives in a distinct clade (Figure 1B). Indeed, phylogenetic analysis revealed that strain SKN60^T^ formed a monophyletic cluster with type strains of *S. roseicoloratus* TRM 44457^T^ and *S. laurentii* ATCC 31255^T^.” (Line number 277-283) as well as throughout the revised version.

11. The identification results of Menaquinone were not presented in the article.

- As suggested, we have mentioned the results of Menaquinone as “The menaquinone analysis showed presence of MK-9.” (Line 269).

12. Only the "antiSMASH 3.0 database tool" is introduced in the Materials and Methods, and the "antiSMASH 7.0 software" mentioned in line 323 is not introduced in the Materials and Methods.

- We are sorry for the oversight. In our study, we have used the antiSMASH 7.0 version and rectified the antiSMASH tool 3.0 with antiSMASH 7.0 as “Biosynthetic gene cluster (BGC) encoding for the production of secondary metabolites was analysed using antiSMASH 7.0 database tool (Blin et al. 2019).” (Line 157-158).

13. Line 439, "terrae" requires italics.

- We have italicized the word "terrae" as “*Streptomyces terrae* (terʹrae. L. gen.n. *terrae* pertaining to soil, referring to isolation source)” (Line 472).

14. Line 442, "upto" should be changed to "up to".

- Modified as suggested by the reviewer as “It tolerates up to 8% NaCl” (Line 475).

15. Line 445, "iso C15:0" should be changed to "iso-C15:0", "iso C16:0" should be changed to "iso-C16:0", "C17:0 3OH" should be changed to "C17:0 3-OH". It is also recommended to make the same changes in other areas.

- The suggested changes are made in the revised manuscript: “Major cellular fatty acids are iso-C_15:0_, anteiso-C_15:0_, iso-C_16:0_ and iso-C_17:0_ 3-OH” (Line 478) as well as throughout the revised draft.

16. Line 447-448, "The type strain is SKN60T (MTCC 13163T, JCM 3577T) isolated from" should be changed to "The type strain SKN60T (MTCC 13163T, JCM 3577T) is isolated from".

- Revised as suggested by the reviewer “The type strain SKN60^T^ (MTCC 13163^T^, JCM 3577^T^) is isolated from an arable subsurface soil sample.” (Line 480-481).

Reviewer: 2

Comments to the Author

The manuscript entitled "An antimicrobial thiopeptide producing novel actinomycetes Streptomyces terrae sp. nov., isolated from subsurface soil of arable land" by Choksket et al, isolated and identified a novel bacterial strain from genus Streptococccus, showed its potential to produce the antimicrobial thiopeptide. Altogether, the study has scientific merit, but I am not sure whether its good fit for fems microbe journal. I have some concerns pertaining to the content of this article mentioned as follows:

- We thank reviewer for the positive feedback and suggestions to improve the quality of our manuscript. We have provided clarifications to all concerns raised by the reviewer.

1. The introduction (lines 77-80) states that the objective was to identify the microbe responsible for producing thiopeptides, while in the results section (line 217), the aim was to screen the rhizosphere soil sample for microorganisms exhibiting antimicrobial activity. However, the methodology lacks a section that specifies whether the isolation of isolates was based on thiopeptide-specific selection or other antimicrobial criteria. This deficiency highlights a major flaw in the experimental design or the hypothesis derived from the results. Therefore, it is necessary to address this issue to ensure the accuracy and reliability of the study.

- We are sorry for the ambiguity. The aim of the study was to identify the microbes producing antimicrobials including antimicrobial peptides. As suggested by the reviewer, we have mentioned the details in introduction and also experimental procedure is included in the revised manuscript as following:
- These antimicrobial peptides are an emerging class of antibiotics against drug resistant Gram-positive bacteria. Since species of the genera *Streptomyces* and *Nocardia* are primarily reported to produce such antimicrobials (Bagley et al. 2005; Bauman et al., 2021), in this study, we have made an attempt to isolate and identify antimicrobial substances including antimicrobial peptide producing strains of these genera (Line 87-91).
- **Soil sample collection and isolation of actinomycetes:** Soil sample was collected from subsurface (20 cm depth) environment of an arable land in Chandigarh and processed immediately to screen microorganisms exhibiting antimicrobial activity. Briefly, 1g of soil was serially diluted in 10 ml of sterile saline and 100 µl aliquots of each dilution (10^-2^ to 10^-4^) were inoculated on to starch casein agar (Himedia, India) plates. The plates were incubated at 30ºC for 3-4 days and observed after every 24 h time interval for antimicrobial producing strains. Colonies showing inhibitory zone in their periphery were picked up and streaked on streptomyces agar (Himedia, India) for further purification, and stored as glycerol stocks in -70 ºC deep freezer until further use. A strain designated as SKN60 was selected for detailed studies. (Line 94-101).

2. It is suggested to add a few lines outlining the isolation protocol in line 83.

- As suggested by the reviewer we have included the isolation protocol in methods:
- **Soil sample collection and isolation of actinomycetes:** Soil sample was collected from subsurface (20 cm depth) environment of an arable land in Chandigarh and processed immediately to screen microorganisms exhibiting antimicrobial activity. Briefly, 1g of soil was serially diluted in 10 ml of sterile saline and 100 µl aliquots of each dilution (10^-2^ to 10^-4^) were inoculated on to starch casein agar (Himedia, India) plates. The plates were incubated at 30ºC for 3-4 days and observed after every 24 h time interval for antimicrobial producing strains. Colonies showing inhibitory zone in their periphery were picked up and streaked on streptomyces agar (Himedia, India) for further purification, and stored as glycerol stocks in -70 ºC deep freezer until further use. A strain designated as SKN60 was selected for detailed studies. (Line 94-101).

3. Please add a statement in line 98 describing the method used to generate the consensus 16S rRNA gene sequence from the Sanger dataset.

- We have elaborated the method to generate the consensus 16S rRNA gene sequence from the Sanger dataset as: “The genomic DNA of SKN60 was extracted using Zymo DNA isolation kit and used for 16S rRNA gene amplification. Briefly, 16S rRNA gene was amplified using the primers 27F and 1492R and 5x firepol master mix (SolisBioDyne, Estonia) in a 50 µl reaction volume. The amplified product was run using 1% agarose gel and purified using HiYield™ Gel/PCR DNA mini kit (Real Biotech Corporation). The purified amplicon was sequenced using three internal primers including 27F, 786F and 1492R (10µl reaction volume) and the sequencing was performed on an ABI Prism 3700 automatic DNA sequencer (Applied Biosystems). The nucleotide sequence. ab1 files were analysed using FinchTV version (1.4.0) software and assembled manually. The consensus sequence was used for BLAST sequence similarity search on EzTaxon server (Chun et al. 2007). (Line 123-131).

4. Please provide more details on how Quast was used to identify the conserved genes as I am not aware it does and add a reference for BUSCO.

- Our apologies for this mistake. We thank reviewer for raising this point and agree that QUAST is used for quality assessment of the genome assemblies. We have modified the text in revised manuscript as:
- “De-novo assembly was performed using ABySS (1.2.10), SPAdes (3.1.30) and Velvet (1.2.150) (http://www.usadellab.org/cms) version 3.13 (Bankevich et al. 2012). Quality assessment of the genome assemblies was performed using QUAST 4.0 version (Gurevich et al. 2013) which provides better statistics for velvet assembly and the presence of assembled genes were checked using BUSCO V2 (Simao et al., 2015).” (Line 141-146).

5. In line 232, please include the range, optimum temperature, pH, and NaCl tolerance values.

- As suggested, we have mentioned the physiological conditions as “Growth was observed up to 40ºC temperature with optimum growth at 30ºC and pH tolerance up to 10.” (Line 260-261).

6. It is recommended to use the 16S rRNA gene from the genome, rather than the one generated from the Sanger sequencing method, to construct the phylogenetic tree. Sanger sequencing should only be used for isolate identification.

- We agree with the reviewer. We have extracted the 16S rRNA gene from genome sequence and used for analysis. All details are mentioned in revised manuscript: “Further, 16S rRNA gene sequence retrieved from genome sequence (1532 bp) was relatively longer and identical with sequence obtained from Sanger method, and thus, it was used for similarity search and phylogeny. The maximum sequence identity was observed with type strains of *S. laurentii* ATCC 31255^T^ (99.31%), *S. roseicoloratus* TRM 44457^T^ (99.03%), *S. showdoensis* NBRC 13417^T^ (98.47%) and *S. vietnamensis* GIMV4.0001^T^ (98.31%). The phylogenetic tree constructed using neighbour-joining phylogenetic analysis showed strain SKN60^T^ forming a cluster with closest phylogenetic relatives in a distinct clade (Figure 1B).” (Line 275-281 and Figure 1B).

7. In line 252, please state the genome size in Mb and include the bp in the N50 contig size value.

- As suggested by the reviewer, we have provided the details in revised manuscript as follows: “The whole genome sequence of *Streptomyces* sp. strain SKN60^T^ contained 7.8 Mb assembled in 87 contigs (with pegs) with N50 contig size of 312 247 bp.” (Line 285-286).

8. Line 255 should be removed as it is part of the method, not the result.

- We have removed the details of sequence method from result and added in Material and Methods section.

9. Please revalidate the ANI and AAI results based on comment 6.

- As suggested by the reviewer we have reanalyzed the ANI and AAI with closely related strains as suggested by the reviewer. “ANI analysis of genome sequences displayed maximum identity with *S. roseicoloratus* TRM 44457^T^ (88.45%) *S. showdoensis* NBRC 13417^T^ (87.02%), *S. laurentii* ATCC 31255^T^ (86.42%) and *S. vietnamensis* GIMV4.0001^T^ (85.91%) (Supplementary Figure S2A). Similarly, dDDH values also shown in descending order 33.7, 30.7, 29.2 and 28.3% with its closest strains *S. roseicoloratus* TRM 44457^T^*, S. showdoensis* NBRC 13417^T^, *S. laurentii* ATCC 31255^T^ and *S. vietnamensis* GIMV4.0001^T^, respectively (Supplementary Figure S2B). The descending order of AAI values with closely related species showed 85.61, 83.52, 80.71% identity with *S. roseicoloratus* TRM 44457^T^, *S. showdoensis* ATCC 15227^T^ and *S. laurentii* ATCC 31255^T^, respectively (Supplementary Figure S2C).” (Line 292-300)

10. Line 285 needs to be clarified.

- We are sorry for the confusion and the sentence is modified as suggested by the reviewer: “The genome sequence of strain SKN60^T^ was visualized in right order using Proksee server.” (Line 315).

11. In lines 289 and 291, the pangenome and core genome are two separate concepts and should not be confused. The paragraph should be modified to avoid unnecessary confusion for readers.

- We agree with the reviewer and pan-genome and core genome are mentioned separately in revised manuscript: “The pan-genome analysis in terms of protein coding genes of 30 *Streptomyces* species genomes revealed 46047 ortholog clusters from 162482 total genes that comprised pan-genome. The sum of unique genes was clustered into 24930 gene clusters. The core genome contained 1568 gene clusters (Figure 2C). The increase in total genomes decreased core genome size, which is stabilized with about 1580 genes after the addition of 27^th^ genome indicating the closure of core genome. The core-pan plot revealed increase in pan-genome with the addition of new genomes and therefore, it is considered as an open pan-genome.” (Line 318-325)

12. What does "ca" mean in line 327?

- “ca” is an abbreviation of “Circa,” (Latin word that means something approximate). We have modified the sentence as “The predicted cluster contained 17 ORFs (spans approximately 30 kb) encoding all genes” (Line 358-359).

13. In line 359, what does "0 h" refer to? Is it a zero time point?

- Yes, it is 0 h time point. Sentence modified to avoid confusion “haemolysis with respect to the positive control (1% Triton X100) observed at 0 h time point” (Line 391).

14. The manuscript appears to be written in a format suitable for classification and taxonomical characterization for a specific journal type. However, it may need to be reformatted for FEMS Microbe journal or audience.

- As suggested by the reviewer we have formatted the manuscript as per FEMS Microbes journal guidelines.

**Reviews of manuscript FEMSMC-2023-009.r1**

**Reviewer 2: Roshan Kumar, Magadh University, Post-Graduate Department of Zoology**

**Recommendation: Accept**

The changes has been made as per previous comments.

**Reviewer 3**

**Recommendation: Accept**

The manuscript has been significantly updated by the authors, and it is now acceptable in its current form.
